# Instruments for assessing pain in persons with severe dementia

**DOI:** 10.1590/S1980-57642014DN82000003

**Published:** 2014

**Authors:** Natália Lindemann Carezzato, Gabriela Gallego Valera, Francisco Assis Carvalho Vale, Priscilla Hortense

**Affiliations:** 1 Resident Nurse in Nursing in Health of Adults and Elderly - UNICAMP, Campinas SP, Brazil. Bachelor and Licentiate degree in Nursing from the Federal University of São Carlos, Center for Biological Science and Health/Department of Nursing, São Carlos SP, Brazil.; 2 Nurse, Master’s in Nursing. Center for Biological Science and Health/Department of Nursing - Federal University of São Carlos, São Carlos SP, Brazil.; 3 Medical Doctor, PhD in Neurology/Neurosciences, Associate Professor. Center for Biological Science and Health/Department of Medicine - Federal University of São Carlos, São Carlos SP, Brazil.; 4 Nurse, PhD in Nursing, Associate Professor. Center for Biological Science and Health/Department of Nursing - Federal University of São Carlos, São Carlos SP, Brazil.

**Keywords:** pain, dementia, pain measurement, validation studies

## Abstract

Through an integrative literature review involving the CINAHL, Cochrane, Embase, LILACS, PsycINFO, PubMed databases, tools available in the literature for assessing pain in individuals with severe dementia were identified along with versions validated for use in Brazil. We found 1501 relevant articles which, after selection of abstracts and full reading, yielded a final sample of 33 articles. The analysis enabled the identification of 12 instruments: ABBEY PAIN SCALE; ADD; CNPI; CPAT; DOLOPLUS-2; MOBID and MOBID-2; MPS; NOPPAIN; PACSLAC; PADE; PAINAD and PAINE. Despite the wide variety of tools for assessing pain in individuals with severe dementia worldwide, it was observed that only four are available in Portuguese, of which two are culturally adapted for Brazilian Portuguese (NOPPAIN and PACSLAC) and two validated for Portuguese of Portugal (DOLOPLUS and PAINAD), pointing to the need for further validation of instruments for use in Brazil.

## Abstract (pt)

Por meio de revisão integrativa de literatura, realizada nas bases de dados CINAHL, Cochrane, Embase, LILACS, PsycINFO, PubMed, foram identificados os instrumentos disponíveis na literatura que avaliam a dor em pessoas com demência grave e quais desses instrumentos têm versão validada no nosso país. Foram encontrados 1501 artigos, e, após seleção dos resumos e leitura na íntegra, a amostra final constituiu-se de 33 artigos. A análise possibilitou identificar 12 instrumentos: ABBEY PAIN SCALE; ADD; CNPI; CPAT; DOLOPLUS-2; MOBID e MOBID-2; MPS; NOPPAIN; PACSLAC; PADE; PAINAD e PAINE. Apesar de ampla variedade de instrumentos de avaliação de dor em pessoas com demência grave no mundo, observou-se que apenas quatro instrumentos estão disponíveis em português, sendo dois adaptados culturamente para o português brasileiro (NOPPAIN e PACSLAC) e dois instrumentos validados para o português de Portugal (DOLOPLUS e PAINAD), o que sugere a necessidade de validação de mais instrumentos no nosso país.

## INTRODUCTION

Pain is defined as "An unpleasant sensory and emotional experience associated with actual or potential tissue damage, or described in terms of such damage", characterized by the context and perception of its meaning.^1^

In the course of dementia, sufferers may no longer interpret sensations, often because they are unable to recall their pain or verbally communicate it to their caregivers. In view of the definition for pain^1^ and considering it is a subjective experience, the non-verbalization of pain further hampers its detection and measurement, rendering the assessment of pain a challenge.^2-7^

In moderate and severe dementia processes, non-verbal expressions and behavioral changes become more frequent, some of which may indicate pain symptoms. In these cases, social withdrawal, aggressivity, psychomotor agitation or mood swings may be signs of the presence of pain.^8,9^ The absence of reports of pain should not be interpreted as the absence of pain in elderly patients with cognitive impairments. Thus, evaluations of pain must be undertaken.^10^

Another aggravating factor is that behavioral changes in patients with severe dementia can often be regarded by health professionals as symptoms of cognitive or psychiatric decline, leading to a neglect of the diagnosis and treatment of pain in older adults with dementia.^5,10,11^ Conversely, attribution of pain systems to manifestations of restlessness, agitation or aggressivity may induce inappropriate over prescription of antipsychotics, tranquillizers, sedatives and other psychotropic drugs.

Prompted by this worrying scenario, studies have sought solutions toward improved management of pain in this fragile population in the form of devising specific instruments for identifying and measuring pain in non-communicative patients.^7^ These tools are essential in the clinical setting for identifying interventions and efficacy of strategies, thereby preventing erroneous interpretation by professionals and resulting in better management of pain.^9,10^

In view of this need, measuring instruments are key elements in refining the communication interface between those feeling and those treating pain. It is clear that the success of assessing pain in elderly with dementia hinges on the development and implementation of an adequate instrument for use in this population.^11^

Based on this perspective, the aim of the present study was to identify the instruments available in the literature assessing pain in persons with severe dementia and to determine which of these instruments have Portuguese versions validated for use in Brazil.

## METHODS

An integrative review of the literature was carried out entailing five stages: identification of the relevant question; search and selection of relevant articles; categorizing of studies (data collection); analysis and interpretation of the data; summarizing of the knowledge gleaned.^12,13^ Performed between September 2012 and December 2013, the integrative review of the literature was based on the following constraining questions: "What instruments are available in the literature assessing pain in persons with severe dementia?" and "Which of these instruments assessing pain in persons with severe dementia are validated for the Portuguese language?". A search of articles of interest was performed on the following electronic data-bases: CINAHL, Cochrane, Embase, LILACS, PsycINFO and PubMed.

The descriptors chosen for searching the articles were extracted from the DeCS (Descriptors in Health Sciences) and from the MeSH (*Medical Subject Headings*), and were "demência" (dementia) and "avaliação da dor" (assessment of pain) for searches on LILACS and "*pain*", "*dementia*", "*cognitive impairment*", "*evaluation studies*", and "*validation studies*" for searches on CINAHL, Cochrane, Embase, PsycINFO and PubMed.

The selection of articles covered all publications available up to 2013. The inclusion criteria adopted were: articles whose abstracts indicated the study of instruments for assessing pain in persons with severe dementia; original articles in human subjects aged 18 years or older with a medical diagnosis of severe dementia; articles written in English. Portuguese or Spanish languages. The exclusion criteria adopted were: articles whose full versions were not available online or in libraries to which the researchers had access.

All articles selected were read in full. After the reading process, data collection based on the defining question was performed, followed by summary and analysis of the data collected on the tools and their validation.

## RESULTS

A total of 1501 articles were retrieved. Duplicate studies found in more than one database, or by different cross-references of descriptors, were included only once. Thus, of the articles originally retrieved and subsequently pre-selected for final reading, a total of 33 articles remained (Table 1).

**Table 1:** Results of database searches according to cross-references between descriptors and by stage of selection of scientific output investigating instruments for assessing pain in persons with severe dementia.

Database	Cross-reference operations	Articles retrieved (n=1501)	Articles selected by abstract (n=125)	Duplicate articles excluded (n=61)	Articles read in full (n=64)	Articles selected (n=33)
CINAHL	Pain AND Dementia AND Validation	164	36	23	13	6
	Pain AND Dementia AND Evaluation					
	Pain AND Cognitive impairment AND Evaluation					
Cochrane	Pain AND Dementia AND Validation studies	468	7	0	7	0
	Pain AND Dementia AND Evaluation studies					
	Pain AND Cognitive impairment AND Evaluation					
EMBASE	Pain AND Dementia AND Validation study	76	7	3	4	1
	Pain AND Dementia AND Evaluation					
	Pain AND Cognitive defect AND Evaluation					
LILACS	Demência (Dementia) AND Avaliação da dor (Pain assessment)	344	30	0	30	22
PsycINFO	Pain AND Dementia AND Validation	156	19	13	6	1
	Pain AND Dementia AND Evaluation					
	Pain AND Cognitive impairment AND Evaluation					
PUBMED	Pain AND Dementia AND Validation studies	293	26	22	4	3
	Pain AND Dementia AND Evaluation studies					
	Pain AND Cognitive impairment AND Evaluation					

Analysis of the results obtained in the literature review revealed 12 instruments, published between 1999 and 2012, shown in Table 2.

**Table 2:** Adaptation/validation studies of instruments for assessing pain in persons with severe dementia by author/year, journal of publication, instruments assessed/acronym, instrument language, and confirmed psychometric tests.

Author/Year	Instruments	Instrument language	Confirmed psychometric tests
Kovach et al., 1999^14^	• Assessment of Discomfort in Dementia (ADD)	English	• Inter-rater Reliability
Warden et al., 2003^15^	• Pain Assessment in Advanced Dementia (PAINAD)	English	• Internal Reliability •Construct Validity
Villanueva et al., 2003^16^	• Pain Assessment for the Dementing Elderly (PADE)	English	• Internal Reliability •Inter-rater Reliability •Test-Retest Reliability •Construct Validity •Criteria Validity
Fuchs-Lacelle; Hadjistavropoulos, 2004^17^	• Pain Assessment Checklist for Seniors with Limited Ability to Communicate (PACSLAC)	English	• Inter-rater Reliability •Internal Reliability •Concurrent Validity
Abbey et al., 2004^18^	• Abbey Pain Scale	English	• Apparent Validity •Content Validity •Concurrent Validity •Internal Reliability •Inter-rater Reliability
Holen et al., 2005^19^	• DOLOPLUS-2	Norwegian	• Criteria Validity;
Hutchison et al., 2006^20^	• Pain Assessment in Advanced Dementia (PAINAD)	English	• Not performed;
Cohen-Mansfield, 2006^21^	• Pain Assessment in Noncommunicative Elderly persons (PAINE)	English	• Internal Reliability •Inter-rater Reliability •Test-Retest Reliability •Criteria Validity
Zwakhalen et al., 2006^22^	• Pain Assessment in Advanced Dementia (PAINAD) • Pain Assessment Checklist for Seniors with Limited Ability to Communicate (PACSLAC) • DOLOPLUS-2	Dutch	• Internal Reliability •Intra- and Inter-rater Reliability •Convergent Validity
Leong et al., 2006^23^	• Pain Assessment in Advanced Dementia (PAINAD)	English	• Divergent Validity •Concurrent Validity
Horgas et al., 2007^24^	• Non-Communicative Patient’s Pain Assessment Instrument (NOPPAIN)	English	• Intra- and Inter-rater Reliability •Convergent Validity
Holen et al., 2007^25^	• DOLOPLUS-2	Norwegian	Test-Retest Reliability •Inter-rater Reliability
Zwakhalen et al., 2007^26^	• Pain Assessment Checklist for Seniors with Limited Ability to Communicate (PACSLAC)	Dutch	• Internal Reliability
Husebo et al., 2007^27^	• Mobilization-Observation-Behavior-Intensity-Dementia Pain Scale (MOBID)	English	• Internal Reliability •Inter-rater Reliability •Apparent Validity •Construct Validity
Schuler et al., 2007^28^	• Pain Assessment in Advanced Dementia (PAINAD-G)	German	• Internal Reliability •Inter-rater Reliability
Costardi et al., 2007^29^	• Pain Assessment in Advanced Dementia (PAINAD-I)	Italian	• Internal Reliability •Inter-rater Reliability •Test-Retest Reliability •Concurrent Validity •Construct Validity
Cervo et al., 2007^30^	• CNA* Pain Assessment Tool (CPAT)	English	• Not performed
Mahoney et al., 2008^31^	• Mahoney Pain Scale (MPS)	English	• Inter-rater Reliability •Internal Reliability •Construct Validity •Concurrent Validity •Clinical Feasibility
Ferrari et al., 2009^32^	• Non-Communicative Patient’s Pain Assessment Instrument (NOPPAIN)	Italian	• Inter-rater Reliability •Concurrent Validity •Convergent Validity
Cervo et al., 2009^33^	• CNA* Pain Assessment Tool (CPAT)	English	• Inter-rater Reliability •Test-Retest Reliability •Internal Reliability •Apparent Validity •Construct Validity •Criteria Validity •Clinical Feasibility
Husebo et al., 2009^34^	• Mobilization-Observation-Behaviour-Intensity-Dementia Pain Scale (MOBID)	English	• Intra- and Inter-rater Zreliability
Pickering et al., 2009^35^	• DOLOPLUS^®^	ItalianPortuguese (PT)EnglishSpanishDutch	• Test-Retest Reliability •Inter-rater Reliability
Takai et al., 2010^36^	• Abbey Pain Scale (J)	Japanese	• Internal Reliability •Test-Retest •ReliabilityCriteria ValidityConstruct Validity
Lin et al., 2010^37^	• Pain Assessment in Advanced Dementia (PAINAD-C)	Chinese	• Inter-rater Reliability •Internal Reliability •Test-Retest Reliability •Content Validity •Construct Validity
Ersek et al., 2010^38^	• Checklist of Nonverbal Pain Indicators (CNPI) • Pain Assessment in Advanced Dementia (PAINAD)	English	• Internal Reliability •Inter-rater Reliability •Construct Validity
Ando; Hishinuma, 2010^39^	• DOLOPLUS-2	Japanese	• Inter-rater Reliability •Apparent Validity
Chen et al., 2010^40^	• DOLOPLUS-2	Chinese	• Internal Reliability •Inter-rater Reliability •Construct Validity •Clinical Feasibility
Husebo et al., 2010^41^	• Mobilization-Observation-Behaviour-Intensity-Dementia Pain Scale (MOBID-2)	English	• Test-Retest Reliability •Inter-rater • Reliability • Internal Reliability • Apparent Validity • Construct Validity • Concurrent Validity
Lorenzet et al., 2011^5^	• Pain Assessment Checklist for Seniors with Limited Ability to Communicate (PACSLAC)	Portuguese (BR)	• Content Validity
Lints-Martindale et al., 2012^42^	• Assessment of Discomfort in Dementia (ADD) • Checklist of Nonverbal Pain Indicators (CNPI) • Pain Assessment Checklist for Seniors with Limited Ability to • Communicate (PACSLAC) • Pain Assessment for the Dementing Elderly (PADE) • Pain Assessment in Advanced Dementia (PAINAD) • Non-communicative Patient’s Pain Assessment Instrument (NOPPAIN)	English	• Internal Reliability •Inter-rater Reliability •Construct Validity
Batalha et al., 2012^43^	• Pain Assessment in Advanced Dementia (PAINAD-PT)	Portuguese (PT)	• Internal Reliability •Inter-rater Reliability •Construct Validity
Zwakhalen et al., 2012^44^	• Pain Assessment Checklist for Seniors with Limited Ability to Communicate (PACSLAC-D)	Dutch	• Clinical Feasibility.
De Araújo; Pereira, 2012^45^	• Non-communicative Patient’s Pain Assessment Instrument (NOPPAIN)	Portuguese (BR)	• Content Validity

* CNA: Certified Nursing Assistant.

The following instruments were identified: *Abbey Pain Scale*, original in English,^18^ translated to the Japanese version;^36^ ADD, original in English;^14,42^ CNPI,^38,42^ original in English;^46^ CPAT, original in English;^30,33^ DOLOPLUS-2,^47^ original in French (DOLOPLUS^®^, first version 1993),^48^ translated to versions in Norwegian,^19,25^, Dutch,^22^ Italian,^35^, Portuguese,^35^ English,^35^ Spanish,^35^ Dutch,^35^ Japanese^39^ and Chinese;^40^ MOBID^27,34^ and MOBID-2,^41^ original in English; MPS, original in English;^31^ NOPPAIN,^24,42^ original in English,^49^ translated into Italian^32^ and Portuguese;^45^ PACSLAC,^42,44^ original in English,^17^ translated into Dutch version in original form,^22^ short version^26^ and into Portuguese;^5^ PADE,^42^ original in English;^16^ PAINAD,^20,23,38,42^ original in English,^15^ translated into version in Dutch,^22^ German,^28^ Chinese,^37^ Italian^29^ and Portuguese from Portugal;^43^ PAINE, original in English.^21^ Summary data outlining each instrument found are given below.

**Abbey Pain Scale.** Assesses vocalization, facial expression, changes in body language, behavioral changes, psychological changes and physical changes. Severity of pain is assessed individually for each of its 6 items.^18,36^

**Checklist of Nonverbal Pain Indicator (CNPI).** Comprising the items vocalization, facial expression, stimulus, friction, agitation and verbal complaints, which are marked as "present" or "absent" under two conditions: in movement or at rest.^38,42^

**Certified Nursing Assistant Pain Assessment Tool (CPAT).** Comprising the categories: facial expression, behavior, mood, body language and activity level. If scoring positive, subsequent assessment of pain is required, where the health professional is responsible for indicating the action to be taken.^30,33^

**DOLOPLUS-2.** Consisting of 10 items, divided into three groups, namely, somatic reaction, psychometric reaction and psychosocial reaction. This instrument assesses the progression of the pain experience.^19,22,25,35,39,40^

**Mobilization - Observation - Behavior - Intensity - Dementia Pain Scale (MOBID).** This instrument assesses nociceptive pain during guided movements of the trunk and extremities. Five items of active movements are observed: opening of both hands, lifting of both arms towards the head, extending and bending of knees and hip joints, rolling to each side and sitting on the edge of the bed. All the movements are performed one at a time gently by the nursing team and stopped immediately if pain behavior is noted. Three indicators of pain behavior are recorded by the nurse: pain utterances, facial expression and defense.^27,34^

**MOBID-2.** Is an extended version of two parts of the MOBID instrument. The first part consists of performing of five guided movement items from the MOBID. The second part includes the reporting by the caregiver on pain originating from the head, mouth and neck; heart, lungs and chest wall, abdomen; pelvis and genital organs, and lastly, the skin.^41^

**Mahoney Pain Scale (MPS).** This instrument comprises an assessment of the items facial expression, breathing, vocalization, body language, signs of agitation in behavior, signs of changes in sleep/appetite, symptoms and changes in vital parameters, and history of pain. Besides assessing the severity of pain, the scale can differentiate between pain and agitation. It also allows pain to be located on a pain map, with patients observed preferably at rest. Raters are instructed to inspect and lightly touch 22 areas marked on a drawing of the human body on a sheet (front and back) and place an "x" alongside points where a behavioral response or signs of pathology were noted.

**Non-communicative Patient's Pain Assessment Instrument (NOPPAIN).** This measures pain based on interpretation of the behaviors the patient expresses. The instrument consists of four sections comprising: nine figures showing situations of daily care; six figures showing pain behaviors; pain intensity for each behavior observed; and a figure of a descriptive scale of subjective intensity. The observer first indicates which care procedures are performed followed by the pain behaviors observed, along with the intensity of each.^24,32,42,45^

**Pain Assessment Checklist for Seniors with Limited Ability to Communicate (PACSLAC).** This is divided into 4 subscales: facial expressions (13 items); body activity/movement (20 items); social/personality/mood indicators (12 items); physiological indicators/feeding/sleep changes, and vocal behaviors (15 items).^5,17,22,26,42,44^

**Pain Assessment for the Dementing Elderly (PADE).** This assessment has three parts: physical (observable facial expression, breathing pattern and posture); global assessment, allowing the caregiver to give an overall pain rating for the patient under their care and for activities of daily living (getting dressed, feeding, transfer from bed to wheelchair).^16,42^

**Pain Assessment in Advanced Dementia (PAINAD).** comprising 5 categories of behavior: breathing, negative vocalization, facial expression, body language and consolability. Each is organized into three subcategories with behavioral descriptors allowing the recognition of the presence of pain or normality.^15,20,22,23,28,29,37,38,42,43^

**Pain Assessment in Noncommunicative Elderly persons (PAINE).** This is a 22-item instrument. The first 15 items are distributed into 4 subgroups: specific motor repetitive behaviors (facial distortions, restlessness, among others), specific vocal repetitive behaviors (moaning, crying, screaming, among others), unusual behaviors (posture, apathy, rigidity, among others) and those related to activities (music, arts, among others). The other seven clinical indicators include falls, trembling, changes in vital signs, edema, blood stains, and broken bones.^21^

**Assessment of Discomfort in Dementia (ADD).** This assessment was devised to recognize and aid the treatment of physical and affective discomfort as well as pain in patients with dementia. The most recent version has 5 categories: facial expressions, mood, body language, voice and behavior. After assessment, recommendations for interventions are provided.^14,42^

A wide variety of instruments were found for assessing pain in persons with severe dementia. However, only two of these instruments have been culturally adapted for Brazilian Portuguese (the PACSLAC^5^ and NOPPAIN^45^) and two previously validated for Portuguese of Portugal (DOLOPLUS^35^ and PAINAD^43^ (Table 3).

**Table 3:** Characterization of instruments adapted and validated for Portuguese (BR, PT) found by integrative review of the literature.

Instrument	Author/Year	Article country/Instrument language	Psychometric test performed on final version?	Application of adapted instrument (N)	Psychometric properties attained
DOLOPLUS	Pickering et al., 2009^35^	France/Portuguese (PT)	Yes	57 elderly with communication disorders, with or without suspected pain and behavioral change were inclusion criteria	ICC: inter-rater=0.95ICC: test-retest=0.95r: inter-rater=0.97r: test-retest=0.99
PACSLAC	Lorenzet et al., 2011^5^	Brazil/Portuguese (BR)	No, only cultural adaption performed	---	---
PAINAD	Batalha et al., 2012^43^	Portugal/Portuguese (PT)	Yes	99 elderly incapable of self-assessment (with or without dementia)	Cronbach’s a =0.84ICC: inter-rater=0.89Total variance=61.1%
NOPPAIN	De Araújo; Pereira, 2012^45^	Brazil/Portuguese (BR)	No, only cultural adaption performed	---	---

BR: Brazil; ICC: Intraclass correlation coefficient; PT: Portugal; r: Pearson’s correlation coefficient.

## DISCUSSION

The instruments found incorporated observational parameters indicative of pain, the most important of which were: changes in facial expression, breathing, vocalization, mood, body language or body movement and activity level. Another less frequent yet important observational parameter indicative of pain was consolability. The studies centered on the criteria of applicability for assessing pain in elderly with severe dementia and on the evaluation of the psychometric properties of the instruments, observed based on the behavior of the subjects assessed.

In fact, identifying pain in individuals with severe cognitive impairment and language deficits involves the collection of different types of information from various sources. The pain behaviors presented by individuals with dementia can vary according to the level of activity.^9^

The majority of the tools found in this study adopted the group of orientations for assessing pain in verbally non-communicative patients,^50^ incorporating six behavioral indicators of pain: facial expressions, verbalizations or vocalizations, body movements, changes in interpersonal interactions, changes in patterns of activity and changes in mental state.

Using measuring instruments to assess pain is a systematic process through which pain is recognized, assessed, documented and reassessed, resulting in improved pain control for all patients, particularly older adults with cognitive impairment. Measuring instruments are key elements in refining the communication between those feeling and those treating pain. It is clear that the success of assessing pain in elderly with dementia hinges on the development and implementation of an adequate assessment tool for use in this population.^11^

In a clinical setting, accurate assessment of pain by measuring instruments, is fundamental for planning appropriate interventions (a key component of health care) and for assessing the efficacy of these intervention strategies. Documenting and formalizing the pain assessment process is essential in the delivery of individualized care, from a legal and professional standpoint, while also eliminates subjectivity.^9,10^

Despite the variety of pain assessment tools for use in individuals with severe dementia worldwide, there are no such instruments for assessing pain in patients with severe dementia in Brazil, pointing to the need for further studies in this area.

Given that pain numbers among the main factors that negatively impact quality of life in elderly with cognitive deficits, the application of specific instruments for effectively measuring, assessing and managing pain is especially important, providing individuals with humane and integral care.
